# A novel 6D-approach to radically transform undergraduate medical education: preliminary reflections from MBRU

**DOI:** 10.1186/s12909-018-1402-0

**Published:** 2018-12-12

**Authors:** Yajnavalka Banerjee, Aida J. Azar, Christopher Tuffnell, Peter J. Lansberg, Riad Bayoumi, David Davis

**Affiliations:** 1 Department of Basic Medical Sciences, Mohammed Bin Rashid University of Medicine and Health Sciences (MBRU), Academic Medical Center, Dubai Health Care City (DHCC), Dubai, 505055 United Arab Emirates; 2 Digital Learning, The Center for Outcomes & Research in Education (CORE), Mohammed Bin Rashid University of Medicine and Health Sciences (MBRU), Academic Medical Center, Dubai Health Care City (DHCC), Dubai, 505055 United Arab Emirates; 3 Department of Pediatrics, Section Molecular Genetics, University Medical Center Groningen, Building 3226, Room 04.14, Internal Zip Code EA12, Antonius Deusinglaan 1, 9713 Groningen, AV Netherlands; 4 Department of Family and Community Medicine, University of Toronto, Toronto, Canada; 5 Masters in Medical Education Program, Department of Medical Education, University of Dundee, Nethergate, Dundee, DD1 4HN Scotland, UK

**Keywords:** Undergraduate medical education, UME, Basic sciences, Medical education, Journal-club, Novel pedagogical technique, Learning-theory, Constructivism

## Abstract

**Background:**

Designers of undergraduate medical education (UME) need to address the exponentially expanding volume and variability of scientific knowledge, where by didactic teaching techniques need to be augmented by innovative student-centric pedagogical strategies and implementation of milieus, where information, communication and technology-enabled tools are seamlessly integrated, and lifelong information gathering, assimilation, integration and implementation is the ultimate goal. In UME, the basic sciences provide a solid scaffold allowing students to develop their personal critical decisional framework as well as define the understanding of normal human physiology, pivotal for the identification, categorization and management of pathophysiology. However, most medical schools confine themselves to “stagnant curricula”, with the implementation of traditional “teacher centered” pedagogical techniques in the dissemination of the courses pertaining to basic sciences in UME.

**Method:**

To tackle the above paucity, we present a novel “6D-Approach” for the dissemination of concepts in basic sciences through mentored journal-clubs. The approach is informed by a teaching principle derived from Constructivism. The technique in which the 6D-approach can be implemented in UME, is shown using an example from a first-year course of Molecular Biology and Principles of Genetics at our medical school. A reflection on the impact of 6D-Approach for students as well as instructors is also presented.

**Result:**

The 6D-approach was positively received by the students and the formal feedback for the course: Molecular Biology and Principles of Genetics, where the approach was repeatedly employed, indicated that students expressed satisfaction with the teaching strategies employed in the course, with ~ 89% of the students in the cohort strongly agreeing with the highest grading score “extremely satisfied”. Further, the approach through the use of mentored journal clubs encourages retention of knowledge, critical thinking, metacognition, collaboration and leadership skills in addition to self-evaluation and peer feedback.

**Conclusion:**

Hence, through the 6D-Approach, our attempt is to initiate, advance and facilitate critical thinking, problem-solving and self-learning in UME, demonstrated by graduating accomplished, competent and safe medical practitioners.

## Background

The complexity of twenty-first century healthcare requires re-thinking current (medical) educational paradigms. In the past emphasis was on gathering facts and understanding the context of these facts: knowledge, presented to students and trainees in textbooks, often taught by lecturers, in large classroom settings in which passive absorption of the shared knowledge was expected. Information or scientific knowledge has expanded at an exponential rate, requiring undergraduate curriculum planners to address the rapidly increasing Velocity, Variability and Volume of data [1, 2]. Didactic teaching techniques need to be augmented by new student-centric pedagogical strategies and implementation milieus, where information and communication technology-enabled tools are seamlessly integrated, and lifelong information gathering, assimilation, integration and implementation is the ultimate goal. Further, it appears that undergraduate teaching requires the incorporation of techniques which the learner will employ at a later stage in his or her career, permitting a strong and clear role for the continuing education leadership in the medical education continuum.

Over the last few decades, medical curricula are shifting towards a more integrative approach, offering activities that augment critical thinking and problem-solving skills into medical education, a need first foreseen by Abraham Flexner. Flexner advocated the concept that formal analytic reasoning, the kind of rational thinking fundamental to the basic sciences especially the natural sciences, should hold precedence in the intellectual training of physicians [3, 4]. In a further iteration of medical education, curricula in Europe, UK and North America, have begun to evolve from a “science based” to a more “systems-based” approach, in which the primary focus is on the development of core competencies beyond the command of knowledge and facts [5, 6].

However, many medical schools globally continue to confine themselves to a so called “stagnant curricula”, with traditional “teacher-centered” pedagogical techniques and an apparent inability, or even resistance to change [7, 8] therefore limiting the initiation and development of critical thinking, problem-solving and associated skills.

In undergraduate medical education (UME), the conventional basic sciences facilitate the so-called ontogenesis of the medical practitioner. They aim to provide a solid scaffold allowing students to develop their personal critical decisional framework as well as define the understanding of normal human physiology, pivotal for the identification, categorization and management of pathophysiology [9].

Further UME should, also encourage activities that augment critical thinking, problem-solving, metacognition, empathy and collaboration skills, to catalyze action and spark innovation. One approach has been that of journal clubs, a staple of continuing education, scholarly meetings in which individuals convene regularly to critically assess/appraise current/recent articles in the scientific literature [10]. Initially conceived by Sir William Osler 1875, the journal club has evolved in the last few decade playing a significant role in continuing education and even in many medical school curricula where journal clubs are instrumental in teaching critical appraisal skills [11]. Linzer et al. established that a journal club–centered curriculum was superior compared to a weekly faculty-managed lecture at teaching the principles of evidence-based medicine [12]. Similarly, a systematic review by Honey et al. of 16 studies, showed improvement in reading habits and critical evaluation skills in the attendees of the journal club [13]. With the evolution of journal clubs, the content and approaches in which journal clubs are conducted have expanded. Journal clubs today, can be categorized into five types: (A) Critical appraisal-based journal club; (B)Evidence-based journal club; (C) Mentored journal club; (D) Student-led journal club and (E) Virtual journal club. Summarized information with regards to the journal club types is presented in Table 1.

**Table 1:** Different types of Journal Club

Journal club type	Description	Reference
Critical appraisal based journal club	Critical appraisal journal clubs, generally deal with reviewing an article, which is usually chosen and assessed by the presenter using a critical appraisal checklist. The foremost drawback with this type of club is that participants may not feel confident in their critical appraisal skills and are therefore reluctant to join in. Additionally, this kind of journal club is directed to experienced adult learners.	Hill A, Spittlehouse C. What is critical appraisal? London: Hayward Medical Communications, 2006.
Evidence based journal clubs	Evidence based journal clubs involve the process of systematically reviewing, appraising, and using clinical research findings to aid the delivery of optimum clinical patient care. The key feature of this journal club includes posing a question followed by carrying out a literature search, and then selecting relevant papers, as well as critical appraisal of the selected article(s > in light of the question posed.	Phillips R, Glasziou P. What makes evidence based journal clubs succeed? Evidence Based Medicine 2004;9:36–7.
Mentored journal clubs	Mentored journal clubs involve the participation of a mentor who helps the presenter to identify appropriate article(s) that fits to address a prespecified scientific question. The mentor also meets with the presenter prior to the journal club to discuss the article(s) and help the presenter with the preparation of the presentation.	Judd S, Antaki F Approach to presenting a clinical journal club. Gastroenterology. 2014; 146(7):1591–3
Student-led journal clubs	Student led journal clubs are voluntary journal clubs where a student presents an article to address a pre-specified scientific/clinical question. These kind of journal clubs are generally organized by students in their residency years.	Funston G. The promotion of academic medicine through student- led initiatives. Int J Med Educ. 2015 Nov 21;6:155–7.
Virtual journal clubs	Any of the above journal clubs when delivered in an electronic format with discussion taking place via e mail and social media is defined as a virtual journal club. The above four journal clubs require protected time for attendees to attend. Virtual journal club overcomes this limitation.	Oliphant R, Blackhall V, Moug S, Finn P, Vella M and Renwick A. Early experience of a virtual journal club. Clin Teach. 2015 Dec;12(6):389–93.

Currently, in UME, pedagogical techniques focus primarily on dissemination of knowledge, but few teaching stratagems aim at providing the medical student with an understanding of how this knowledge can be applied for practicing evidence-based medicine. Further, UME should also provide the foundation for the growth of non-cognitive and metacognitive skills, which is rarely addressed by the teaching strategies implemented in UME, specifically in the delivery of basic science courses. The overarching aim of this research is to design an innovative pedagogical strategy using journal clubs, whereby the student will be informed of the clinical applicability of disseminated knowledge, using published facts and data available in peer-reviewed literature. Such a strategy will also allow instructors to incorporate evidence-based medicine (EBM) teaching, especially in the delivery of basic science courses.

### The 6D-approach using journal clubs to disseminate concepts in basic science courses in UME

**Fig. 1:**
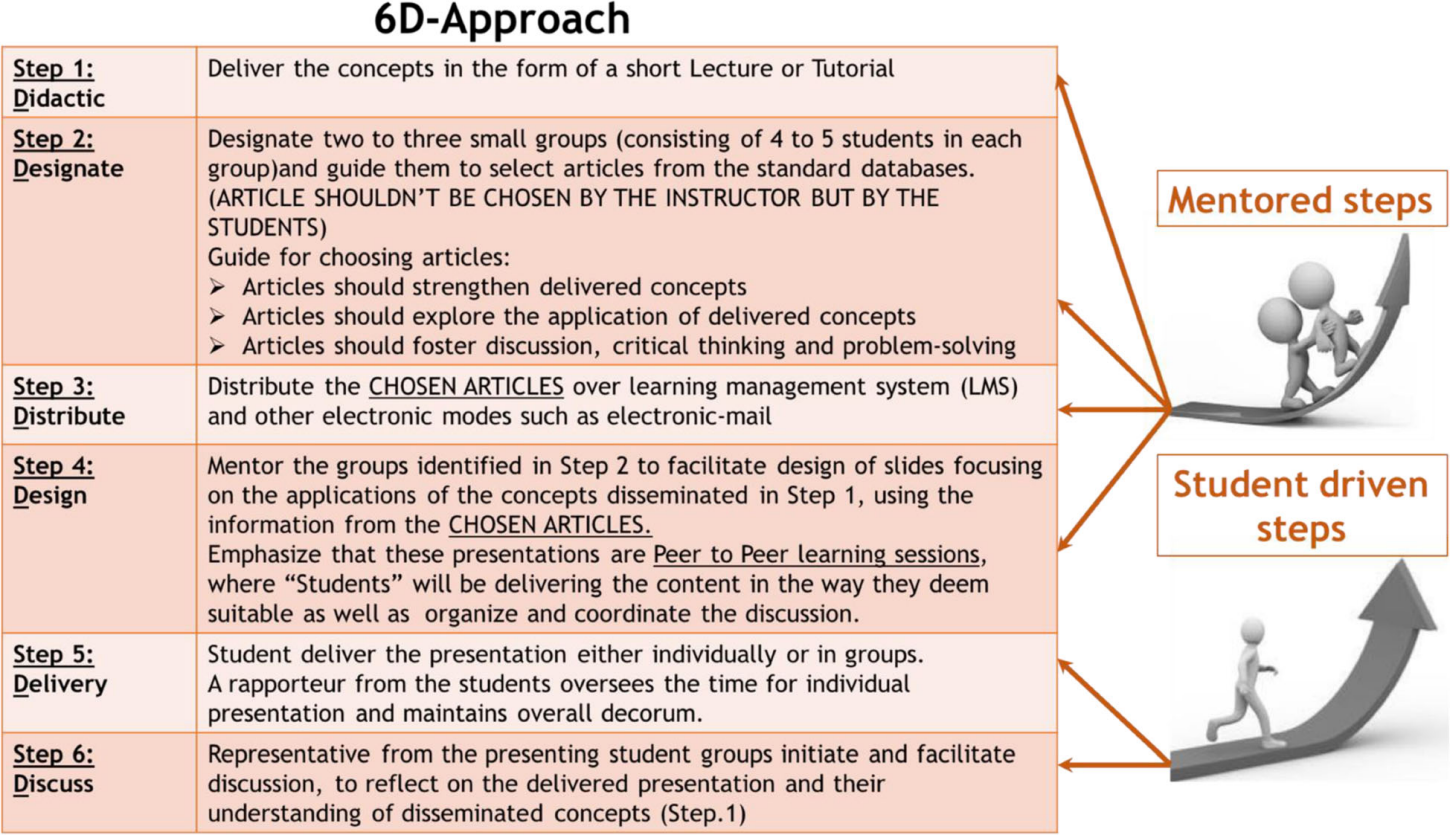
The different steps of the 6D-Approach. (The initial steps are mentor dependent, whereas the concluding steps are student driven)

In this article, we delineate the “6D-Approach” for the dissemination of concepts in basic sciences through mentored journal clubs. The 6-steps of the approach are shown in Fig. 1. The approach is informed by a teaching principle derived from the core educational theory of Constructivism. The technique in which the 6D-Approach can be implemented in UME, is shown using an example from a first-year course of Molecular Biology and Principles of Genetics at our medical school of Mohammed bin Rashid University of medicine and health sciences (MBRU). A reflection on the impact of 6D-approach for students as well as instructors is also presented. Through the 6D approach, our attempt is to initiate, advance and facilitate critical thinking, problem-solving and self-learning in UME, demonstrated by graduating accomplished, competent and safe medical practitioners.

## Methods

### Developing the teaching principle informing 6D-approach

**Fig. 2:**
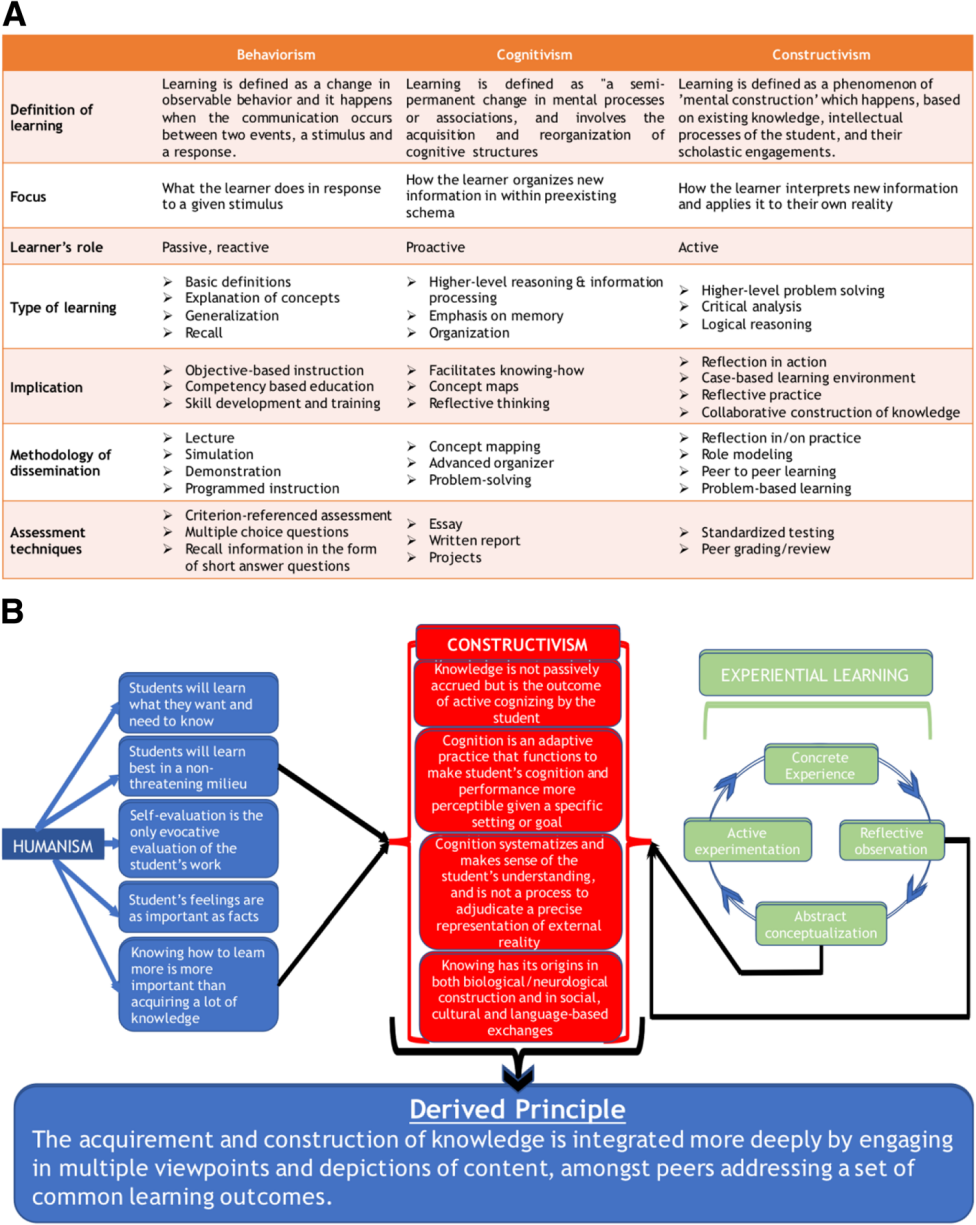
Core educational theories and derivation of the teaching principle informing the 6D-Approach. **(a)** Summary of the three core educational theories. **(b)** Derivation of the teaching principle informing the 6D-Approach. (Although our teaching principle is informed predominantly by Constructivism, we also drew from specific principles of Humanism and Experiential Learning Theories to inform our teaching principle)

The significance of linking instructional approaches or strategies to the theories of human learning has been demonstrated by Onyura et al [14]. Behaviorism, cognitivism, and constructivism are the three core learning theories [15]. These theories differ in how learning is described, which consequently leads to different roles for the learners, and edicts that different teaching schemes and assessment tactics are employed [16]. The key characteristics of the three learning theories are summarized in Fig. 2a.

In order to develop a teaching principle that will inform an approach through which journal clubs can be implemented in UME, we employed Constructivist theory as the foundational core, with contributions from the principles of the Humanistic and Experiential Learning (ELT) theories (Fig. 2b). The principles of Constructivism are reviewed in Fig. 2a. Constructivist theory proposes that definition and understanding are integrated in the conceptual framework of the student, based on existing knowledge, intellectual processing of the student, and their scholastic engagements. The intellectual processing also encompasses reworking, discarding and altering mental schemas that have proved to be unsuitable. Additionally, Constructivism emphasizes that conceptual knowledge cannot be transferred or transcribed from one individual’s brain to another, without the recipient’s brain earnestly participating in the process [17]. In other words, acquisition and creation of knowledge is an evolutionary process where acquisition of knowledge and conception of a mental framework happens as an adaptation to the learning milieu, a concept best summarized by Plotkinism’s key principle “Knowledge is a pervasive characteristic of all of life. It exists in all adaptations in all living creatures” [18].

The philosophical roots of Constructivism are founded on Kantian epistemology where Empiricism (origin of all knowledge is sense experience, which emphasizes the role of experience and evidence in learning) and Rationalism (learning happening through practice or principles of basing opinions and actions on reason and knowledge) are integrated [19]. In terms of psychological origins, Constructivism’s importance is emphasized in the study of Piaget, where the precepts of integration and adaptation have been applied to model the theory of cognitive development [20]. Additionally, doctrines of Constructivism also draw from the dictates of Dewey’s work, where the significance of student’s active participation in the learning process is emphasized [21].

However, we perceived that to apply a teaching principle founded on Constructivism, a positive non-threatening setting is essential, where the student can “think” and “reflect” on the acquired knowledge, aiding learning autonomy and peer-assisted learning. Hence, in designing the principle, we drew from principles of Humanism (learning is student centered and personalized, and the instructor’s role is that of a facilitator) and ELT (learning through reflection on doing) [22, 23]; The overall scheme of the approach is presented in Fig. 2b).

### Teaching principle informing 6D-approach

The acquirement and construction of knowledge is integrated more deeply by engaging in multiple viewpoints and depictions of content, amongst peers addressing a set of common learning outcomes.

### Design and methodology of execution

Employing the derived teaching principle, we designed the 6D-approach. The six steps of 6D-approach (**D**idactics, **D**esignate, **D**istribute, **D**esign, **D**elivery and **D**iscuss) are summarized in Fig. 1; the first four steps are mentored, while the last two steps are student-driven or mentor-independent. The individual steps are elucidated below:

#### Step 1. Didactic

Didactics involves the use of short lectures/tutorials by the instructor to deliver the key concepts with regards to a specific topic. The ideal lecture provides the following: a limited (five or less) main learning objectives; employing effective visuals, analogies, demonstrations, and illustrations to reinforce the main points; making the material available to students prior to delivery; highlighting and summarizing the learning objectives and major points of the lecture clearly.

#### Step 2. Designate

First, the instructor designates two to three small groups (of 4 to 5 students depending on the number of articles to be presented and discussed. While designating the groups the instructor ensures diversity amongst students in each group, since cultural, linguistic and ethnic diversity offers a powerful learning resource in terms of influencing the way students engage with the subject matter of the course, and help them build on these perspectives to augment both their cognitive and non-cognitive skills [24]. Additionally, each group needs to assess their experience as a team to facilitate the development of collective intelligence. To facilitate this, we have designed a rubric (Table 2), providing the student-group with a guideline. The designated groups are introduced to article search and selection strategies by the instructor. While implementing the 6D-approach, we employed the method of Ecker et al [25]. Simultaneously we introduced the students to the PubMed Tutorial homepage created by the National Library of Medicine: http://www.nlm.nih.gov/bsd/disted/pubmedtutorial/and the PubMed help page http://www.ncbi.nlm.nih.gov/bookshelf/br.fcgi?book=helppubmed&part=pubmedhelp.

**Table 2:** Rubric for the assessment and management of student-teams’ experience

Using the Benchmarks identify the behavior that best represents your team’s experience. Address the identified deficiencies through open discussion amongst your team members					
Benchmarks	10	8	6	4	2
Attendance	All, members in the group are present for all meetings, are punctual, and stay for the entire duration of the meeting.		Most members attend majority of the scheduled meetings. When members are away, they inform the team or a designated team- member.		One or more of the members are recurrently absent for the scheduled meetings and do not inform the team, or a designated team-member. Also members are often late or leave early, when a meeting is in progress.
Establishing rational goals	When suitable, rational, and measurable goals are agreed upon and acknowledged and the entire team shares the common objectives/ purpose.		Team members share some objectives but a mutual commitment may be absent.		Defined objectives are missing; members are not tuned to the task or purpose of the group.
Accountability for Results	The obtained result is recognized as a collective-effort.		Team-members work on separate sections of the project and link to each other through a coordinator in the team		Team-members lack coordination with other members of the team and work on the different sections individually.
Communication	Team members communicate with other members openly and treat each other with respect.		Although, mutual respect amongst team- members exists, opinions of specific members aren’t considered while formulating decisions.		Communication amongst team members is limited.
Decision Making	Most decisions in the team are through a consensus.		Majority and minority of decisions prevail at times, when decisions are made by the group.		Decisions are made by specific members and do not reflect that of the team.
Adjusting	The team is able to amend and adjust plans as needs arise.		The team is mostly able to amend and adjust plans as needs arise.		The team lacks focus and lacks the ability to adjust and amend plans.
Assessment of the team	Members regularly assess the progress of the project and appraise the cohesiveness of the team.		Members occasionally assess the progress of the project and there are occasional dissensions amongst team members.		The members never appraise the functioning of the team as a group.
Conflict management	Conflict(s) is/are managed through open discussions amongst team members.		Conflicts are occasionally addressed through open discussions.		Conflicts are never addressed.

#### Step 3. Distribute

Following article selection by the groups, the selected article(s) are made available to other students in the cohort by uploading through the learning management system (LMS) software [26]. This concludes the **D**istribute step. The article(s) could similarly be circulated using electronic-mail or shared over a cloud service, such as Google-Drive, OneDrive, iCloud etc.

An alternative approach is to employ a social media application to host the journal club online, such as that described by Topf et al [27, 28].

#### Step 4. Design

This step focuses on the design of presentation by the student groups. While implementing 6D-approach at MBRU, we recommended the students to follow the guidelines of Schmatlz et al., while designing their presentation [29].

#### Step 5. Deliver

The designated student-group(s) delivers the presentation, during which a representative from the group may deliver the entire presentation or different sections of the presentation are delivered by different group members. While it is important that the instructor leave the choice of the presentation style to the students, groups are instructed to be mindful of time constraints and to ensure interactivity with other learners. At MBRU one of the students from the cohort volunteered as the timekeeper to make sure all presentations started and concluded within a given time-frame.

#### Step 6. Discuss

As in journal clubs at more advanced levels, it is important to ensure that students have time to discuss the article(s), evaluate its strength and weaknesses and to assess if the concepts delivered in **D**idactic step provided them with a suitable background to allow critical appraisal. While implementing the 6D-Appoach we encouraged the student who is moderating the discussion to have a list of initiating and follow-up questions to promote discussion.

## Results

### Validation of 6D-approach through implementation at MBRU for UME

I. *Choosing the course*: We decided to implement the 6D-approach at MBRU for which we selected the course: Molecular Biology and Principles of Genetics, offered in semester 2 of the first-year medical curriculum at MBRU.
  The reason we chose this course is, because with the rapid integration of molecular biology and genetics into medicine it has become evident that practicing physicians as well as medical students and clinical researchers, need to be updated on the new developments especially with important findings based on molecular-biology/genetics related biomedical research. The course provides a foundation for understanding the relationship between molecular biology, developmental biology, genetics, genomics, bioinformatics, and medicine over a period of 15 weeks. One of the key aims of this course is to develop explicit associations between basic research, medical understanding, and the perspective of patients. After attending the course, the students should be able translate clinical understanding into analysis at the level of the gene, chromosome and molecule. Further, the topics in the course encompass concepts and techniques of molecular biology and genomics, and the strategies and methods of genetic analysis, including an introduction to bioinformatics.
II. *Logistics*: The entire cohort consisting of 54 students registered for the course, and there were no dropouts over the duration of the course. Seven presentation sessions were organized throughout the course. Students were informed of the dates of presentation at prior to the commencement of the course. On the day of commencement of the course, a designated student representative was instructed to distribute the students into groups, each group containing 3 to 4 students.
  The representative divided all 54 students into 14 groups. The first 12 groups had 4 students in each group (12 X 4 = 48 students) and the last 2 groups had 3 students in each group (2 X 3 = 6 students). In each presentation session, 2 student groups delivered their presentation.
III. *Overview of a typical session*: Each student group had 14-days, following the Didactic step (Step 1 of 6D-approach) to decide on the article and prepare for the presentation. Slides corresponding to each presentation were uploaded on the LMS and shared amongst the students at least a day prior to the presentation. Each student group was allotted a maximum time of 25 min to deliver the presentation. This was followed by a question/answer or discussion session of 15 min. As overview of the implementation of 6D approach for the topic titled “DNA Replication and Repair” is shown in Table 3. The **D**idactic step focused on defined objectives which were delivered through two lecture sessions. At the end of the, second lecture session the instructor identified two topics for exploring the application of the concepts delivered.
  *Group 1* was Designated to present two case reports with regards to genetic disorders where the DNA repair mechanisms are impaired. These included (A) Fanconi Anemia and (B) Xeroderma pigmentosum.
  *Group 2* was Designated to review the topic “Exploitation of DNA repair mechanisms in cancer therapy”.
  The student articles were Distributed through LMS.
  Each group Designed their presentations separately, with intermittent help and clarifications from the instructor. The designed presentations were then uploaded and shared among all students using LMS.
  Both Groups 1 and 2 Delivered their presentation on a pre-decided day, with other students and the instructor as the audience.
  Following the Delivery of the presentation a student-initiated Discussion session was organized, which critically appraised the articles. The Discussion step of the approach also explored the importance of the concepts delivered in the Didactic step, with regards to their clinical relevance. Case in point, in the Didactic step students were informed that nucleotide excision repair (NER) is the most flexible of all DNA repair mechanisms because the mechanisms ability to eliminate a multifarious structurally unrelated DNA lesions. However, the Discussion step of the approach elaborated that the common denominator of the different types of damage induced by the numerous chemicals to which NER-deficient cells are sensitive seems to be the generation of bulky base adducts which lead to a distortion in helical structure and DNA chemistry relating it to the diseased state Xeroderma pigmentosum.

**Table 3:** Implementation of 6D-Approach in a UME course

Steps of 60- Approach	Topic: DNA Replication and Repair
Step 1. Didactic	Lecture summary: This lecture reviews the mechanism of DNA replication and also the ways in which DNA can be repaired, if any replication error(s) occur. Further the lecture explores, selected causes of DNA damage, and the consequences if the repair mechanisms are impaired. Lecture objectives: Following the lecture students should be able to: 0 Describe the structure-function of the replication machinery in eukaryotes 0 Summarize the key events in the process of DNA repair 0 Identify the different causes of DNA damage 0 Discuss the consequences of failure of DNA repair mechanisms
Step 2. Designate	Two student groups were designated: 0 Group 1 (4 students) presented TWO case reports pertaining to autosomal disorders where repair mechanisms are impaired A. Fanconi’s anemia B. Xeroderma pigmentosum. 0 Group 2 (4 students) presented the topic: Exploitation of DNA repair mechanisms in cancer therapy Both groups performed an extensive literature search and selected the following articles: 0 Group 1 selected TWO articles: (A) For Fanconi’s anemia: The Fanconi Anemia and Breast Cancer Susceptibility Pathways, N Engl J Med. 2010 May 20; 362(20): 1909–1919. doi:10.1056/NEJMra0809889; (B) For Xeroderma pigmentosum: Xeroderma pigmentosum: a case report and review of the literature, J PrevMed Hyg 2010; 51: 87–91 0 Group 2 selected ONE article: For Exploitation of DNA repair mechanisms in cancer therapy: DNA repair mechanisms in cancer development and therapy, Front Genet. 2015 Apr23;6:157. doi: 10.3389/fgene.2015.00157. eCollection 2015.
Step 3. Distribute	The articles were uploaded on the LMS and shared with all the students in the cohort
Step 4. Design	0 Each group had 14 days for the preparation of presentation. 0 Both groups used Microsoft power-point to prepare their slides for presentation. 0 The time, venue and duration of individual presentation was indicated by the instructor to individual groups prior to the commencement of the course.
Step 5. Delivery	0 Both groups delivered the presented their slides in the form of oral presentation 0 All the group members in both Groups 1 and 2 participated in the presentation process in a predetermined sequence (designated by the students in a group) 0 A student from the cohort volunteered to act as the timekeeper during the event 0 Individual groups were allocated 25 min for presentation delivery and 15 min for discussion
Step 6. Discuss	0 Representative from each group facilitated discussion 0 Discussion focused on three aspects: A. Suitability of the article in addressing the assigned topic B. General critic of the different aspects of the article C. Correlating the concepts delivered in Step 1, with the findings/data reviewed in presented articles

### Evaluation of 6D-approach

In the present work, our focus was on the design and implementation of the 6D-Approach, an elaborate evaluation of the outcome of the 6D-Approach is still pending and will be addressed in our future work through the design of defined rubrics and questionnaires, employing software module such as Survey Monkey. Hence, the evaluation of the 6D-Approach presented here is only preliminary.

The approach was evaluated informally following Pendleton’s approach [30]. A measure with regards to the approach’s facilitation in knowledge retention was obtained by reviewing the final grade of the students at the conclusion of the course. As the 6D-Approach was implemented repeatedly in the course, we also reviewed the student-feedback obtained at the end of the course.

The 6D-apporach was received positively by the students, who exhibited enthusiasm in both organizing and in participating in the event. In each of the seven sessions the students were informally queried with regards to two questions: (A) What were the strengths of the session? (B) What aspects of the session they believed required attention/improvement? Along, with the students the instructor also addressed the above questions on site.

Few points of note observed by the instructor during the sessions:

- Students from different academic backgrounds effectively functioned as a group.
- Reading habits of students improved significantly from the commencement to the conclusion of the course as was observed through the increase in the depth and content of the questions posed by the students during Discussion. This observation which is in line with the findings of Honey et al [13].

Specific limitations that need to be addressed:

- The time allocated for the Discussion was insufficient in all seven sessions. Since these events are scheduled in line with a designated time-slot, one way to address this deficiency will be to host online discussion sessions over Twitter, Snap Chat, WhatsApp etc.
- Students had difficulty in accessing specific journals (as they weren’t subscribed by the institution). One of the ways to bypass this limitation is to encourage students to refer to articles in open access journals, albeit of repute, as not all open access journals are stringently reviewed [31, 32].

Formal student feedback for the course, like other courses offered during the semester, was obtained online, using a MBRU approved questionnaire identical for all courses. The formal feedback for Molecular Biology and Principles of Genetics indicated that students expressed satisfaction with the teaching strategies employed in the course, with ~ 89% of the students in the cohort strongly agreeing with the highest grading score “extremely satisfied”.

With regards to the facilitation of retention of knowledge following the implementation of the 6D-approach, we appraised the final grades of the students. The course had two-formative and one summative assessments. The pass mark and excellence for individual assessment was determined by Angoff-method [33]. Out of 54 students, 52 (96%) passed and 2 (4%) failed, producing a very high success rate.

## Discussion

The 6D-approach provides a novel strategy to improve UME. Under the following subheadings, we explore the different cognitive and non-cognitive skills that are beneficially affected, when this approach is implemented.

### Deep vs surface learning

**Fig. 3:**
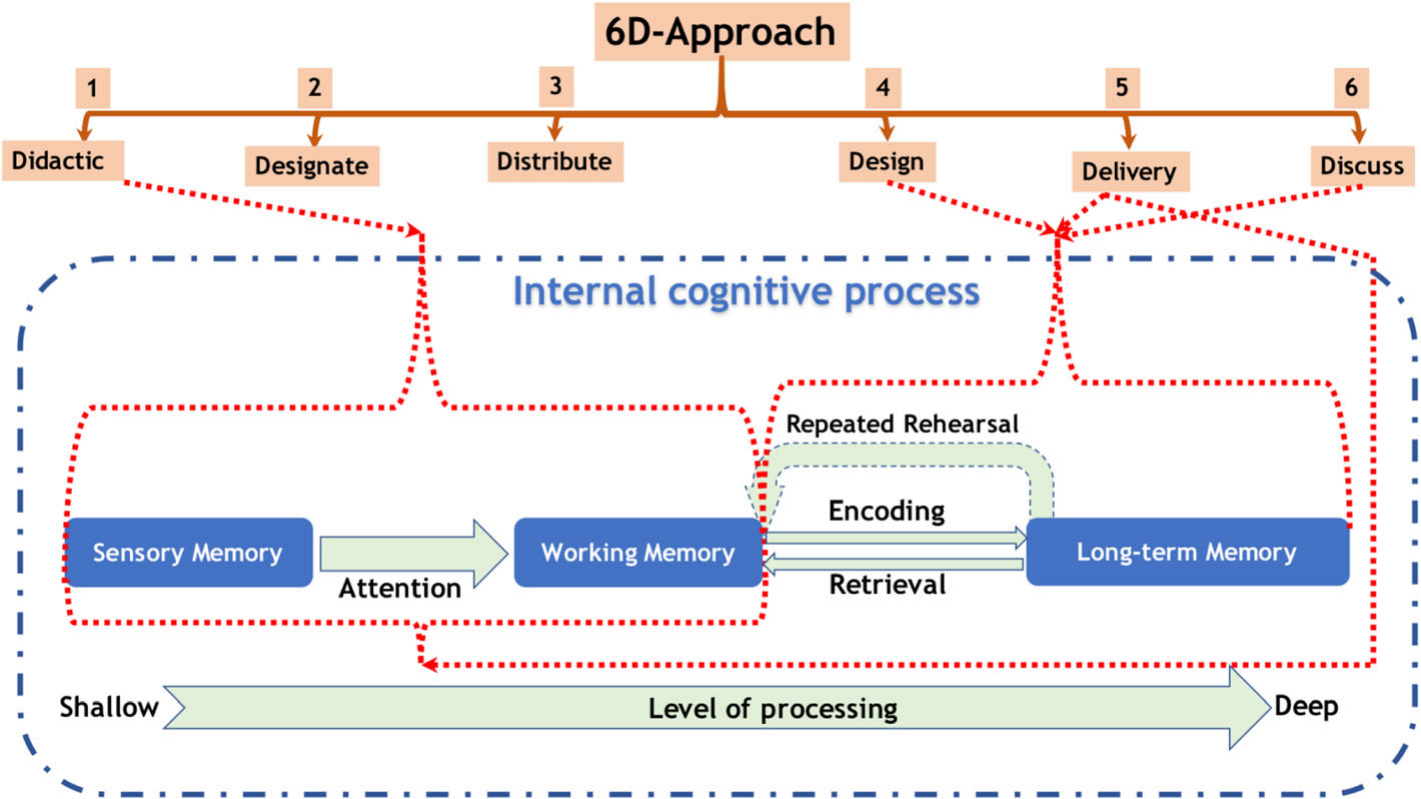
The effect of 6D-Approach on internal cognitive process. (The Design and Delivery steps of 6D-Approach promote elaborate rehearsal, which facilitate Deep-Learning by potentiation of long-term memory)

Didactic teaching rarely facilitates potentiation of long-term memory [34]. When students go-over lecture materials or tutorial problems, maintenance rehearsal or rote memorization occurs leading to “Surface-learning” [35]. In the 6D approach in Steps corresponding to **D**esign and **D**elivery, elaborate rehearsal is facilitated, accelerating “Deep-learning” [35], as the student repeatedly applies the concepts delivered in the **D**idactic step, in analyzing the details of the article as well as reflecting on the data presented in the article in light of the concepts delivered. (Fig. 3).

The exercise of rote memorization is more passive and leads only to short-term retention, whereas elaborative rehearsal is an active learning practice advantageous for transmitting the information into long-term memory [36]. Unlike working memory, long-term memory is limitless in capacity and stores information perpetually in forms of systematized schemas [37]. The 6D approach addresses this goal as repeated retrieval and encoding of data is required in the **D**esignate, **D**esign, **D**elivery and **D**iscuss steps of the approach, which is illustrated in Fig. 3.

### Aiding metacognition

Metacognition is awareness and control of one’s learning or the knowledge and thinking about one’s own or another’s thoughts, feeling, and values [38]. It can be catalogued into: knowledge of cognition and regulation of cognition. Knowledge of cognition relates to what individuals comprehend about their own knowledge base. Regulation of cognition denotes to a set of essential skills that help students control their learning, including planning, monitoring, and evaluation [39].

The 6D approach allows the student to consolidate information in the articles according to a definite concept map. The student designs this concept map, during which s/he requires to assimilate the concepts, which helps in the development of the understanding of “how to learn” not only “what to learn”.

### Encouraging autonomy in learning

Learning autonomy refers to the student’s ability to set learning goals and take charge of his or her learning [40]. The perception of autonomy is founded on three principles: perceived internal locus of causality, volition, and perceived choice. The 6D approach supports the three principles of perception of autonomy. In the 6D-approach, the student selects the article and presents it, in line with the first principle of perception of autonomy. **D**esign, **D**eliver and **D**iscuss steps allow the student independence to design, deliver and appraise the presentation/article in a non-threatening environment amongst his peers. Additionally, these steps also allow the student to set his/her own learning and evolvement goals, addressing the second and third philosophies of learning autonomy.

### Promoting critical-thinking

Critical thinking is an indispensable cognitive aptitude for the individuals involved in different healthcare domains [41]. The 6D approach builds up student’s critical thinking skill by providing a reliant teaching-learning environment wherein **D**esign, **D**eliver and **D**iscuss steps encourage reasoning and analytics, problem solving abilities and welcome new ideas and opinions. Case in point, during Discussion following Group 1’s presentation, one of the students indicated that CRISPR technique of gene editing could be used to effectively treat Fanconi anemia. This new thread of information was not indicated in the article and provided a new dimension to the discussion.

### Development of leadership skills

Larry L. Mathis once commented “*Nothing in a doctor’s medical education qualifies him to be a leader*” [42]; Yet, physicians are expected to be a leader, to bear accountability, and to provide pivotal medical decisions facing a heterogeneous environment. Therefore, it is imperative that activities exploring group dynamics, that promote the development and cultivation of leadership skills are implemented in UME. These include activities focusing on team-leadership abilities, relationship management, emotional intelligence, situational leadership, and the capacity for reflection. The **D**esignate, **D**esign, **D**eliver and **D**iscuss steps of 6D-approach, help train students in the above competencies.

### Facilitating self-evaluation and peer feedback

Self-evaluation and peer-feedback are essential obligations of medical professionals in the improvement and continuance of professional competencies [43, 44]. In the 6D approach, the **D**esign step allows the students to reflect on the concepts delivered in the **D**idactic step. This helps them to self-evaluate their gaps in learning. The **D**iscuss step allows the students to involve in an elaborate discussion with their peers, during which they receive a detailed feedback with regards to not only the presentation but also on their understanding of the concepts with respect to the topic being addressed through the presentation. Although, we did not obtain a formal feedback from the students, we did informally ask them to evaluate the presentations of their peers following Pendleton’s approach of obtaining feedback [30]. This approach emphasizes on the following aspects:

1. Ask the student what went well
2. Tell them what went well
3. Ask the student what could be improved
4. Tell them what could be improved

### Benefits to the instructor in implementing 6D-approach in UME

The 6D approach provides the instructor with opportunities to inculcate novel pedagogical techniques in the curriculum. One of our future aims will be to employ technology enhanced learning (TEL) through the 6D approach. TEL can be defined as an innovative pedagogical approach, that aims to combine learning design and leverage digital technologies to deliver active and engaging, student centered learning [45]. Our endeavor will focus on the effectiveness of one specific social media tool (Twitter) in terms of its application of the 6D approach and impact on groups of undergraduate medical students. It will assess how the medium supports the online group discussion, the efficiency of article dissemination, the selection of articles for the active in-classroom discussions, how group members deal with resistance to participation and over contribution. Conclusions will be drawn around the areas of participation, over saturation of information and the building of a community of practice amongst the group of ‘digital natives’.

#### Practice points

Designers of UME need to address the exponentially expanding volume and variability of scientific knowledge, where by didactic teaching methods need to be augmented by innovative student-centric pedagogical strategies. Such pedagogical strategies need to be informed by focused teaching principles, and should integrate information, communication and technology-enabled tools to promote lifelong information gathering, assimilation, integration. One of these approaches is to employ mentored journal clubs, which are scholarly congregations in which individuals convene regularly to critically assess/appraise current/recent articles in the scientific literature, especially in basic science courses in UME, as these courses provide a platform allowing students to develop their personal critical decisional framework as well as define the understanding of normal human physiology, pivotal for the identification, categorization and management of pathophysiology. Such journal club integrating pedagogical strategies will encourage retention of knowledge, critical thinking, metacognition, collaboration and leadership skills in addition to self-evaluation and peer feedback.

#### Limitations of the present study

We successfully implemented the 6D-Approach in UME, and the approach was favorably received by the students. However, our study has specific limitations:

1. Due to the exploratory nature of this educational approach, we decided to publish and share our approach before we embarked on a larger more formally structured program. We therefore are unable to present a well-designed and executed evaluation of the expected improvements in knowledge retention as well as enhanced non-cognitive skills and competences.
2. We explored the benefits of the 6D-Approach in a course on Molecular Biology and Principles of Genetics. The nature of the chosen topic is characterized by mechanistic and structural aspects of medical science. Topics that require the development of other skills and competencies such as analytical prowess, emotional intelligence and emphatic aptness could improve by implementing the 6D-Approach. However, we cannot extrapolate the results from our pilot project to courses of a distinctly different nature.
3. The positive feedback received from the course participants are an indication that the implementation of the 6D technique can incite enthusiasm in students. However, a formal and more robust measurable outcome need to be formulated and analyzed as to provide an overall accepted scientific proof of the expected superior end results.
4. The successful implementations of the 6D approach builds on the technical and practical implementation of the model. However, like all teaching situations, success depends on the enthusiasm, knowledge and communication skills of the course directors - teachers as well.

## Conclusion

The 6D-approach through the use of mentored journal clubs encourages retention of knowledge, critical thinking, metacognition, collaboration and leadership skills in addition to self-evaluation and peer feedback. Given the significance of the above traits in medical education, the approach provides a strategy to those who want to implement and strengthen the above traits in UME. Finally, and importantly, mentored journal clubs employed in the implementation of the approach represent an example of the medical education continuum, in particular highlighting the use of a continuing education method in UME.

## Abbreviations

6D-approach: (Didactics, designate, distribute, design, delivery and discuss)
MBRU: Mohammed Bin Rashid University of Medicine and Health Sciences
TEL: Technology enhanced learning
UME: Undergraduate medical education
